# The exclusionary approach to consciousness

**DOI:** 10.1093/nc/niad022

**Published:** 2023-10-05

**Authors:** Marlo Paßler

**Affiliations:** Berlin School of Mind and Brain, Humboldt-Universität zu Berlin, Luisenstraße 56, Berlin 10117, Germany; RTG 2386 “Extrospection”, Humboldt-Universität zu Berlin, Luisenstraße 56, Berlin 10117, Germany; Institute of Philosophy, Otto-von-Guericke University, Zschokkestr. 32, Magdeburg, Sachsen-Anhalt 39104, Germany

**Keywords:** methodology, neural correlates of consciousness, contrastive method, exclusionary approach, operationalization problem

## Abstract

The standard approach in the field of consciousness research involves identifying the neural correlates of consciousness (NCCs) by comparing neural activity between conscious and unconscious trials. However, this method has been met with criticism due to the lack of consensus on how to operationalize and measure consciousness. In this paper, I propose an alternative approach: the exclusionary approach. Rather than utilizing near-threshold conditions to contrast conscious and unconscious trials, this approach leverages the widely accepted notion that subjective reports are reliable under normal conditions. I propose that this can be done by assessing whether consciousness remains stable across trials while manipulating other factors such as reports, tasks, stimulation, or attention. We can use the resulting contrast to exclude certain kinds of neural activity as candidate NCCs. This method produces results that are less contentious, allowing for the establishment of hard criteria for theories of consciousness. Additionally, this approach does not require the development of new research paradigms, but can incorporate existing studies, particularly those aimed at identifying confounding factors in the standard approach. It is important to note, however, that the proposed exclusionary approach does not negate the value of the identification approach. Rather, they should be considered as complementary methods.

## Introduction

Consciousness is at the heart of the mind–body problem and keeps fascinating and puzzling philosophers, scientists, and laymen alike. For most of its history, the mind–body problem was discussed on the metaphysical level with dualists arguing for a fundamental difference between the mind and the body and monists arguing that there is no such difference. However, both sides agreed that we are conscious. From the first-person point of view, it seems not difficult at all to define and almost ridiculous to deny one’s own consciousness. Nagel puts it this way: “fundamentally an organism has conscious mental states if and only if there is something that it is like to be that organism - something it is like for the organism” (Nagel 1974). The idea of “what-it-is-likeness” has become the minimal common denominator for phenomenal consciousness and defines the target phenomenon of consciousness science. Thus, phenomenal consciousness, henceforth just “consciousness” or “experience,” provides a clear and tangible target from a first-person perspective. “It begins when we wake in the morning from a dreamless sleep and continues until we fall asleep again, die, go into a coma, or otherwise become ‘unconscious’” (Searle 2000). Right now, you have countless sensations that make it feel like something to be you for you. You not only see words on a white background, but you also feel the chair beneath you, your breath coming in and going out of your lungs, hear cars or birds from a distance, and many other experiences that together define what it is like to be you for you at this moment. Whether you experienced all of that before I directed your attention to it is a central topic of debate. Nevertheless, if asked, you can give a rough sketch of how all of that feels to you. If asked what it is like to have a dreamless sleep or to be anesthetized, on the contrary, it seems like there is nothing to give a sketch about. In our quest for scientific understanding, we must be careful not to oversimplify these phenomena by replacing them with more easily measured, functionally defined components, as this could divert us from the true objective of consciousness research.

The contrastive analysis established by Baars (1986) is the standard approach for trying to pinpoint the neural events and processes underlying such feelings and experiences. Despite the intimate relationship we all have with our own consciousness, it has proven to be a notoriously difficult task to operationalize it into measurable terms (Irvine 2012; Phillips 2018b; Carruthers et al. 2019; Bayne and Shea 2020; Michel 2020, 2021). This is due to the fact that the reliability of introspective reports deteriorates under near-threshold conditions, which are the relevant conditions for the standard approach. The emerging flaws of reports in these cases have led to a number of competing methods (Overgaard 2015), many pointing toward different and dissociable processes (Dehaene et al. 2006; Charles et al. 2013; Lamme 2020). The detrimental result of this incoherence is that almost every part of the brain is currently considered as a neural correlate of consciousness (NCC) by at least some studies (Yaron et al. 2022). I call the set of all these candidate NCCs the “borderland” because it is not clear yet if these candidates are truly related to consciousness.

In this paper, I propose a strategic shift from identifying to excluding NCCs: the exclusionary approach. The goal of this approach is to narrow down the set of candidate NCCs on consensual grounds by exploiting the intimate relationship we have with our own consciousness under normal conditions. It should be noted, however, that identification and exclusion are not exclusive but complementary tasks. Respectively, the exclusionary approach can draw heavily on the literature revolving around the identification of confounding factors in standard NCC research, as highlighted by Overgaard (2004) or Aru et al. (2012b). They propose paradigms that aim to distill the “true” or “proper” NCCs, henceforth distilling paradigms. By introducing three constraints—the consciousness scope constraint, the vividness constraint, and the scaffolding constraint—exclusionary paradigms (read as “distilling under these constraints”) can bypass difficult debates because they only make use of report-based methods in a way regarded trustworthy even by dark pessimists and skeptics. The results can thus be used as hard criteria for all theories of consciousness. That is, theories that predict NCCs that have already been excluded on a consensual basis must be either adapted or rejected. The paper is divided into two main parts. The first part, “The borderland of consciousness science,” critically looks at the standard way of studying and identifying NCCs. The identification approach tries to pinpoint NCCs by isolating consciousness as a variable. The second part, “From identification to exclusion,” proposes an alternative way of thinking about consciousness research. Instead of trying to pinpoint the brain activity correlated with consciousness, this approach focuses on ruling out candidate NCCs by dissociating them from consciousness. This approach is more practical because it allows us to approach consciousness under optimal conditions as it only depends on consciousness being stable across trials, rather than varying in isolation under near-threshold conditions. It is argued that the results of exclusionary paradigms are acceptable even by skeptics under certain constraints, granting them the status of hard criteria applicable to all theories of consciousness.

## The borderland of consciousness science

In the next sections, I introduce the standard approach for identifying NCCs and their limitations. The standard approach—established by Baars (1986)—aims to pinpoint the neural events and processes underlying conscious feelings and experiences by comparing brain activity of minimally different conscious and unconscious trials. We also explore the literature surrounding the identification of confounding factors in NCC research, highlighting the challenges and limitations of the standard approach. However, operationalizing consciousness into measurable terms has proven to be challenging and potentially undermines our efforts, with different studies utilizing different methods and pointing toward dissociable neural patterns and processes. As a result, almost every part of the brain is being considered as a candidate NCC at the moment, leading to a lack of hard bottom-up criteria for theories of consciousness. This abundance of candidate NCCs, referred to here as the “borderland,” raises questions about the feasibility of the traditional NCC project.

### The standard approach in NCC research

A simple definition of NCCs is provided by Koch (2004, 16): “the minimal set of neural events sufficient for a specific conscious percept.” [Chalmers David (1995, 1996 and Chalmers (2000) were the first to properly define NCCs and did so in more sophisticated ways than above to make sure that NCC research both tracks consciousness and bypasses difficult debates about what he called the “hard problem” of consciousness. There still is a lively and interesting debate about how precisely NCCs need to be defined to ensure this (e.g. Fink 2016). Here, I stick with the more accessible definition by Crick and Koch because the focus here is on methodology.] The goal of NCC research is to identify these minimal sets of neural cells and activity patterns that elicit a specific conscious experience. In theory, “[s]timulating the relevant cells with some yet-to-be-invented technology that replicates their exact spiking pattern should trig-ger the same percept as using natural images, sounds, or smells” (Koch 2004, 16). Furthermore, if this technology also had the capability to inhibit all other cells while keeping the firing rates of the relevant cells constant, the same specific conscious percept should still be triggered. This highlights the importance of the “minimal” aspect of NCCs. For example, while stimulation of the retina leads to conscious percepts, if the same specific percepts can also be triggered by stimulating other cells while silencing retinal ones, it follows that the retina is not part of the NCC. However, currently, we do not possess the technology to stimulate and inhibit individual cells at will. Therefore, the question emerges, what methods do we have at present to investigate consciousness?

One of the obvious tools that can be used to investigate consciousness is brain imaging techniques, such as functional magnetic resonance imaging (fMRI), electroencephalography, magnetoencephalography, and electrocorticography. These techniques allow scientists to depict the neural activity that occurs in the brain when a person consciously perceives a stimulus. However, it is challenging to determine from brain images alone which specific neural patterns are minimally sufficient for a particular conscious percept, as the brain is a complex system. That is, every conscious processing of stimuli is accompanied by a plethora of processes that are not strictly necessary for the respective experiences to occur. When using brain imaging techniques it is thus important to consider how to ensure that the target process, the NCC, is isolated such that no other processes are depicted in the brain image.

In 1986, Baars introduced the contrastive method as a thoroughly empirical approach to study consciousness using brain imaging (Baars 1986) and it soon became and still is the standard method in NCC research (Dehaene and Changeux 2011; Lepauvre and Melloni 2021). The fundamental idea was “to specify the minimal conditions of occurrence and nonoccurrence of a conscious experience, as expressed in a reliable way by subjects. One can do this by contrastively analyzing closely matched pairs of psychological events which seem to differ only in respect to the fact that one member of the pair is conscious, while the other is not” (Baars 1986). Although the paradigms used in contrastive analysis vary in several respects (Kim and Blake 2005), the general idea is the same. A target stimulus is presented at near-threshold or bistable conditions, where it alternates between conscious and unconscious processing while other parameters remain constant, and the differential neural activity between conscious and unconscious trials then is taken to depict the minimal conditions of occurrence and nonoccurrence of the experience in question.

Two common paradigms used to create near-threshold conditions in which the target is sometimes subjectively invisible are masking (Stanislas et al. 2001) and attentional blink (Shapiro et al. 1997; Marois et al. 2000). In the masking paradigm, a target stimulus is presented in close proximity to a task-independent mask surrounding the target location which interferes with the processing of the target. By changing the temporal proximity of the mask, the difficulty of consciously perceiving the target can be manipulated. In the attentional blink paradigm, participants are presented with a rapid stream of stimuli and asked to identify specific target stimuli among them. It has been found that the visibility of a second target stimulus is impaired when it is presented within 200 ms after the first target, providing another means to manipulate the visibility of a target stimulus. Upon adjusting the stimulus parameters to ensure the target is consciously perceived in approximately 50% of trials, one can theoretically measure the NCC. This process involves the subtraction of the average brain response from trials where the target was invisible from those where it was visible.

A prominent paradigm for creating bistable perception is binocular rivalry. This captivating perceptual phenomenon arises when two distinct images are presented concurrently to each eye. Rather than integrating the two images into a coherent visual scene, the observer’s conscious perception oscillates between the two images, despite the unchanging nature of the physical stimuli (Blake and Logothetis 2002). Binocular rivalry provides two primary strategies to isolate NCCs.

The first strategy is to contrast periods of perceptual dominance, allowing the identification of regions that alternate with perception compared to those that remain stable despite fluctuating perceptions. For instance, Sheinberg and Logothetis (1997) demonstrated that only 20% of cells in V1 oscillate with changing perception, while up to 90% of cells in the inferotemporal cortex (IT) do so. These results indicate that V1 activity is largely contingent on the actual physical stimulus presented to both eyes, whereas IT activity is more influenced by conscious perception, aligning better with the expected behavior of an NCC.

The second strategy emphasizes the identification of brain regions that instigate the alternations observed during binocular rivalry. This entails contrasting rivalry conditions with non-rivalrous replay scenarios that physically alter to simulate rivalry. For example, only the left image might be presented for a certain duration, followed by the exclusive presentation of the right image. Among others, Lumer et al. (1998) proposed that increased activity in the frontoparietal regions during rivalry, as opposed to replay conditions, implies that these areas play a pivotal role in conscious perception.

However, the application of such rivalry-related activity to locate NCCs is intricate and might be inherently flawed. Specifically, it remains unclear whether the rivalry-related activity maps onto a single mechanism that selects the suppressed representation and brings it into consciousness while simultaneously suppressing the previously dominant one, and if this mechanism is truly integral to conscious perception (Michel 2022b).

This brings us to two general concerns of the standard methodology in consciousness science, which relies heavily on two key assumptions. First, it assumes successful isolation of the target process, and second, that the target process genuinely tracks consciousness rather than some other related process. Both of these assumptions will be critically examined in the next two sections, respectively.

### Distilling the “true” NCCs

The initial excitement surrounding the NCC program began to diminish as researchers became increasingly aware of the fundamental challenges it presented. One of these challenges was inherited from subtractive methods in general. Friston et al. (1996) argue that there is no such thing as “pure insertion” of a target process into the brain, as target processes mutually affect other processes due to the dynamic nature of the brain. This means that any contrast image captures not only the neural events and processes that are relevant to the target process under investigation but also other neural activity that needs to be dissociated from the target process in order to accurately depict it. This dissociation problem poses a significant challenge for NCC research.

Overgaard (2004) suggests that the dissociation problem leads to contradictory findings and a lack of trust in the results obtained through contrastive analysis. To effectively investigate NCCs, it is thus crucial to address these confounding factors. In simple terms, the contrastive method on its own would only work “if one would be able to argue that there are no unconscious processes different when one is, say, watching a stimulus for 10 ms (and thus have no conscious perception of it) and, say, for 300 ms (thus having a quite clear conscious perception of it)” (Overgaard 2004). However, it has become apparent in recent decades that it is much easier to argue against that claim (Melloni et al. 2011; Aru et al. 2012a; Naotsugu et al. 2015; Michel 2017; Nani et al. 2019) than to defend it. Confounding factors present a severe methodological pitfall, but they can also help us to make sense of some of the contradictory results in NCC research.

A striking example is the presence of measured NCCs before stimulus onset, as found in Aru and Bachmann (2009). They show that an increase in certain gamma oscillations is highly predictive of conscious trials, even when present up to 50 ms before stimulus onset. While this technically qualifies as an NCC, in that it appears in the contrast image, it is crucial to consider whether this correlation is a result of the neural events giving rise to the experience, the true NCC, or whether it is merely due to enabling or other probability relations for experiencing the stimulus. Busch Niko et al. (2009) suggest that instead of demonstrating a mysterious form of foresight, it is much more reasonable to assume that gamma oscillations, which are known to represent changes in brain excitability, only affect the probability that a weak, near-threshold stimulus will be consciously perceived. This emphasizes the need to dissociate processes that merely correlate with a specific experience in a given experimental setup from those that are truly minimally sufficient for it. These confounding processes can come in two varieties: those that are active before the proper NCC and those that come after it. Aru et al. (2012a) suggest naming pre-conscious processes “NCC-pr” and post-conscious or consequent processes “NCC-co”.

An example of an NCC-pr is the gamma oscillation just mentioned. However, not all NCCs-pr are created equal, as some may play a more significant role in conscious experience than others. Some more interesting NCCs-pr that have been discussed are attention, prior expectation, or rivalry-related processes in binocular rivalry. Let us consider attention. Some researchers argue that attention is necessary for conscious experience and may even be considered synonymous with it (Posner 1994). However, it may well be that attentional mechanisms are not among the minimally sufficient set for conscious experience. As with the retina, if it were possible to stimulate single brain cells and induce an experience while silencing the areas underlying attention, this would demonstrate that attentional mechanisms are not part of the proper NCC. In the absence of such a technique, we must show that the NCCs can be dissociated from attention, e.g. by demonstrating that they can be manipulated independently. This strategy has proven to be a hard nut to crack, with a number of studies claiming to have demonstrated such a dissociation (Mack and Rock 1998; Lamme 2004; Julian et al. 2018) and others claiming the opposite (Cohen Michael et al. 2011; Molly et al. 2017; Julian et al. 2018). Whether or not attention is constitutively linked to consciousness is still a central debate in consciousness science (Nani et al. 2019) and is related to many important theoretical disputes, such as the overflow debate (Block 2011; Overgaard 2018) or the richness of perception (Kouider et al. 2010; Cohen Michael et al. 2016). Resolving this question would be crucial in many respects, as many theories make explicit assumptions about how consciousness is related to attention (Pitts Michael et al. 2018).

In-between obvious NCCs-pr such as pre-stimulus gamma oscillations and very disputed ones such as attention, there is a broad spectrum of confounding factors. A study by Melloni et al. (2011) sheds light on the practical importance of considering potential confounding factors in the search for the NCC, by demonstrating that neural activity in conscious trials can vary depending on whether conscious perception is driven more by sensory evidence or by prior expectations. To control for this, the researchers manipulated conscious perception in two different ways: by changing the amount of sensory evidence and by previously exposing participants to the images before the test phase. This allowed them to dissociate the influence of prior expectations from that associated with sensory evidence. The results show significant differences in neural activity between the two conditions. Since conscious experience as such should not be affected by whether it is driven more by evidence or by expectation, the researchers argue that prior expectations confound our measures, in particular by shortening the latency of NCCs. This is important for the debate about when NCCs begin to emerge, whether it is 100 ms after stimulus onset (Roeber et al. 2008), 300 ms after (Dehaene and Changeux 2011), or some other time.

On the other hand, NCCs-co refer to post-conscious processes that are automatically triggered by conscious perception or are a result of the experimental design. Recent studies have focused on report-related artifacts as sources of contamination in the results (Naotsugu et al. 2015; Michel 2017). We will discuss them in depth in the second part of the paper.

This literature demonstrates that the search for the physical footing of consciousness is a complex and dynamic endeavor. While significant progress has been made in recent years, the challenge of dissociating true NCCs from confounding factors via distilling paradigms remains a formidable one. As we move forward, the next critical step in consciousness research is addressing the operationalization problem, which is not only concerned with isolating the target process, but judging if we actually target consciousness.

### The operationalization problem

The operationalization problem concerns the conundrum of translating experience into measurable terms such as reports. While many scientific fields successfully operationalized their theoretical constructs, consciousness science might be special in that it is often questioned whether its target phenomenon can ever be operationalized. Chalmers (1995) argues that “bridging principles” are needed in order to overcome the explanatory gap proposed by Levine (1983) that separates phenomenal from functional states. While the idea that consciousness is special has a lot of intuitive appeal, it is still up for debate if this is actually the case (Pauen and Haynes 2021). However, it is undeniable that modern theories of consciousness often rely on some sort of questionable leap of faith in order to justify the methods they use to measure consciousness.

Global workspace theorists, for example, propose that consciousness should be operationalized using reports. It is essential to note that this operationalization is not derived from the global workspace theory (GWT) itself; rather, it is based on the pre-theoretical observation that people can typically report on what they are conscious of, offering a rudimentary but valuable starting point. It is this line of thought that motivates the conceptualization of consciousness as the “publicity organ” of the brain in the first place (Baars Bernard 1997). The idea is that consciousness is the brain’s mechanism for broadcasting information globally and making it accessible to various cognitive systems, including those underlying reports.

Echoing this perspective, Stanislas et al. (2001) assert that “subjective reports are the key phenomena that a cognitive neuroscience of consciousness purports to study”. At first glance, this operationalization seems convincing. But Chalmers (1995) argues that even if we assume that all and only contents of the global workspace are conscious contents, there is still the question of why only the information within the global workspace feels like something. Put differently, global workspace theorists can only assume that the information is experienced because it is globally accessible via a bridging principle, but they cannot explain why. The why question is the fundamental problem that Chalmers (1995) refers to as the “hard problem” and it is one of the reasons why the link between global broadcasting and experience remains vague, leaving leeway to question the underlying operationalization.

In addition to such philosophical considerations, report-based methods have also been the subject of debate in the empirical sciences. One of the main criticisms of this approach is that the reliability of reports deteriorates under near-threshold conditions, as demonstrated by studies such as Bruner Jerome and Postman (1947). They show that all else being equal, inappropriate stimuli have different reporting thresholds than neutral stimuli. This suggests that not only the visibility of a stimulus but also one’s confidence in what one sees plays a role in the decision to report. If decision criteria can be biased, the results of the study may also be biased, calling into question the accuracy of reports as a means of clearly separating unconscious from conscious processing. It is therefore crucial for researchers to consider these potential biases when using reports as an operationalization of consciousness (Spener 2015; Michel 2019; Irvine 2021).

Report-based methods have also been criticized in terms of availability and sufficiency. Availability is a concern because reports require that participants be able to communicate and cooperate with the experimenter. However, in some of the most interesting cases, such as coma patients or animals, verbal reports are not available (Tononi and Koch 2015). Moreover, reports are not always sufficient. For example, in the case of language-based artificial intelligence, verbal reports do not provide a reliable indication of consciousness as discussed in the debate over the relevance of the Turing test for consciousness (Saygin et al. 2000). The same seems to be the case for Anton–Babinski patients, a rare kind of cortical blindness where patients deny vigorously their own blindness even though they are unable to use visual information to guide their behavior (Celesia Gastone and Brigell 2005). In summary, there are a number of limitations and criticisms associated with the use of reports as the sole operationalization of consciousness.

For these reasons, some authors have rejected report-based methods as a means of operationalizing consciousness. Instead, they have turned to the use of objective, bias-free methods (Eriksen 1960). One widely used objective method involves presenting participants with multiple alternatives and forcing them to make a choice, rather than relying on free self-reports. Forced-choice tasks reduce decision bias because participants have to respond even if they are uncertain of what they saw. The idea is that by eliminating the opportunity to refrain from reporting, the choice becomes independent of decision biases.

Objective measures, while free of decision bias, have even more severe limitations when it comes to sufficiency than reports. One of the major problems is that they assume that sensitivity to information is equal to experiencing that information. It is safe to assume that this is not the case for all systems, as bacteria, plants, artificial agents, or thermostats, all can perform above chance in some sense. But even in humans, this assumption is challenged by phenomena like blindsight, where patients are able to perform above chance on visual tasks in a blind field where they deny having any experience of the visual information being processed (Weiskrantz 1990). Furthermore, a study by Lau and Passingham (2006) demonstrates a similar dissociation in healthy individuals. These examples illustrate the limitations of objective measures as the sole operationalization of consciousness.

The investigation and interpretation of divergences between objective and subjective measures of perception, sometimes referred to as “unconscious perception” (Snodgrass et al. 2004), has become a prominent focus in the field of consciousness research (Peters et al. 2017; Phillips 2018a, 2021; Michel 2022a). And it has led to increased efforts to find methods in-between unbiased objective and biased subjective measures, including confidence and metacognitive sensitivity measures which are designed to measure subjective visibility independent of any decision bias (Kunimoto et al. 2001; Koriat 2007; Fleming Stephen and Lau 2014; Michel 2022c). Despite these efforts, there is currently no universally accepted method, and it remains uncertain whether there will ever be a single solution that satisfies all stakeholders.

Furthermore, Block (1995, 2007) has consistently raised concerns regarding the ability of behavioral tasks, regardless of their biases, to capture consciousness. This skepticism not only challenges the accuracy of reports but also their validity as indicators of conscious experience in general. The crux of the problem lies in the fact that reportability and decision criteria can be altered independently of the actual conscious experience. To illustrate this point, we can revisit the classic finding by Bruner Jerome and Postman (1947) that individuals have a lower reporting threshold for the word “shot” compared to its inappropriate cousin. As both words undergo similar visual processing, it is plausible to assume that we also perceive them similarly, but the inappropriate nature of the latter word deters us from noticing and reporting it.

This issue ties into the broader richness debate (Kouider et al. 2010; Cohen Michael et al. 2016), which concerns the question if noticing something equates with consciously seeing something. If there is indeed a dissociation between consciousness and reportability, then our concerns call into question the validity of using report-based methods as a measure of consciousness, especially given how they might misrepresent consciousness in many everyday scenarios where we perceive without noticing because our attention is focused elsewhere. Block’s (2011) “overflow argument” prominently defends this dissociation, suggesting that conscious perception is much richer than cognitive access and therefore a perceptual, rather than a cognitive phenomenon. This view echoes the famous Sperling (1960) study that showed that participants could recall nearly any item from a 3 × 3 matrix when cued to report a specific one shortly after stimulus offset, but only managed to recall an average of four items when asked to report as many as possible after stimulus offset. This discrepancy points to a fragile high-capacity store, referred to as “iconic memory” (Miller George 1956), which Block suggests may be the locus of conscious perception.

Another proponent of the idea that consciousness is not a cognitive phenomenon and independent of the attentional bottleneck is Lamme. He demonstrated that visual processing in the absence of attention can be much more complex than previously thought and that these complex processes depend on recurrent interactions (Jasper et al. 2012), which in turn correlate well with some of the established paradigm cases of consciousness. Lamme (2004) mentions the following findings: backward masking disrupts recurrent processing but not feedforward processing, visibility can be impaired by transmagnetic stimulation that targets only recurrent processing, anesthesia reduces or suppresses recurrent processing but not feedforward processing, and the neural correlates of complex visual functions such as figure-ground segregation are suppressed in attended but unseen stimuli. From this, Lamme concludes that experience is associated with visual rather than cognitive functions and emerges independent of attention (Lamme 2020).

Another approach that relates consciousness to neural, rather than behavioral markers, is integrated information theory (IIT) (Oizumi et al. 2014; Tononi et al. 2016). This theory posits that we can infer certain axioms about consciousness from introspection alone. According to IIT advocates, these axioms are that consciousness exists and is integrated, specific, structured, and definite. From these axioms, a measurement of consciousness, called “Phi”, is formally constructed. If a system has a Phi value >0, it has the proper causal structure to be considered conscious, again regardless of its cognitive or behavioral capacities. However, this theory has been criticized for its lack of empirical support (Cerullo Michael 2015), for being difficult to operationalize in practice (Barrett Adam and Seth 2011), and for its seemingly absurd consequences, such as DVD players being more conscious than humans (see Aaronson’s (2014) blog post: https://scottaaronson.blog/?p=1799).

These operationalizations are only a few examples of the many that have been proposed over the years, and the conflicting claims they raise about each other have led to a resurgence of skepticism. These doubts are no longer purely philosophical but stem from practical challenges that have arisen from conducting research on consciousness. Phillips (2018b) highlights the “methodological puzzle” of determining whether consciousness requires cognition and the difficulty to investigate it, given the current data and methods. Irvine (2021) expresses “dark pessimism” toward the justificatory role of introspective reports when validating measures of consciousness. Michel (2020) adds to these concerns by emphasizing that the field of consciousness science has actually struggled with a lack of agreement about how to operationalize consciousness for >150 years, demonstrating persistent underdetermination. Instead of accepting contradictory findings at face value, scientists often simply reject the underlying operationalizations and detection rules.

This specifies the central challenge for the standard approach: how to identify that one special transition in the processing hierarchy (the minimal condition) responsible for the emergence of a specific aspect of experience. There are a multitude of potential candidates, such as the transition from feedforward categorization to recurrent visual organization (Lamme 2020), the transition from non-integrated to integrated information (Tononi et al. 2016), or the transition from only locally effective to globally broadcasted content (Mashour George et al. 2020). Determining which of these or other candidate transitions represent the minimum condition for a specific experience to arise is essential for uncovering its true NCC. To do this, it is necessary to reach a consensus on which operationalization of consciousness to adopt. In the following section, I demonstrate the negative impact of the current lack of consensus on this matter.

### The borderland of candidate NCCs

In what follows, the term “borderland” is introduced to designate the collection of all the candidate NCCs that are currently discussed in the literature. This borderland is meant to encompass the diverse and often contradictory results of NCC research to serve as a representation of the degree of (dis)agreement within the field. Importantly, the borderland provides a tangible way to evaluate theories of consciousness from a theory-neutral perspective. By determining the extent of candidate NCCs that have been proposed, theories can be tested against them. If a predicted NCC is not in the borderland, the respective theory needs to be adapted or rejected. The extent of the borderland over time should also be a clear, theory-neutral indicator of the progress in the field.

In a first approximation, a recent meta-analysis by Yaron et al. (2022) will form the basis to evaluate the borderland. The following data thus come from Yaron and colleagues’ meta-analysis and their corresponding open-source website (https://contrastdb.tau.ac.il/). In their meta-analysis, they include 412 experiments that have been published between April 2001 and October 2019 in the context of the four leading theories of consciousness, that is GWT, IIT, higher-order theories (HOTs), and recurrent processing theory (RPT). The focus on just these four obviously fails to depict the whole extent of the borderland given the high and ever-increasing number of theories out there, as well as those experiments that do not mention any theory. But for the following, this is rather a supportive than problematic factor. In order to ensure that the difference in findings cannot be explained away easily by differences in modality or kind, I concentrate on studies investigating content-specific NCCs—rather than general or state NCCs—in the visual domain.

Surprisingly, even when restricting the search to studies published between 2001 and 2019, investigating content-specific NCCs in the visual domain only, the borderland includes candidate NCCs across the whole spatial and temporal domain of neural response patterns. In the spatial domain, NCCs have been found across the entire cortex, including 137 experiments in favor of posterior NCCs, 85 in support of parietal ones, and 71 locating NCCs or parts thereof in the frontal areas. In the temporal domain, the most common event-related potentials (ERPs) associated with consciousness are P300 with 50, recurrent processing with 40, and visual awareness negativity (VAN) with 31 supportive experiments. Note that recurrent processing is measured at ∼100 ms, VAN at ∼200 ms, and P300 at 300–400 ms after stimulus onset. When considering findings with <10 supportive experiments, it seems that NCCs are located across the entire temporal and spatial domain (see figures 6 and 7 in Yaron et al. 2022). Furthermore, consciousness has been associated with most of the common oscillatory frequencies, predominantly with gamma (18) and alpha (12).

A possible critique of this assessment is that it is overly broad and exaggerates the extent of the problem. One might argue that over the years, the number of candidate NCCs has been reduced and that it is not fair to include NCCs proposed 20 years ago. However, Yaron and colleagues have investigated this by plotting trends in a time-dependent manner and found that “support for each theory is unaffected by the changing support for other theories, demonstrating a parallel progression of leading theories” (Yaron et al. 2022). More precisely, despite the growing number of experiments supporting each one of the theories, there is no clear trend of one theory gaining more support over time. This indicates a lack of convergence in the empirical data. Along similar lines, few experiments ever provide evidence against a theory. For example, 82% of experiments mentioning GWT are interpreted as supporting it, 87% for RPT, 94% for IIT, and 42% for HOTs [the percentage of HOTs has low representational power due to the small number of experiments mentioning it (*N* = 12)] (see figure 2 in Yaron et al. 2022). It can be concluded that there have been no significant trends toward a specific category of candidate NCCs. Instead, the borderland has remained constant over the years.

It is also important to stress the significant differences among the four major theories in terms of measured NCCs and methods used to measure them (see figure 6 in Yaron et al. 2022 or Seth and Bayne 2022). Studies related to RPT typically focus on the role of “recurrent processing” in occipito-temporal regions, while IIT emphasizes a so-called posterior “hot zone” in occipito-parietal areas. GWT, in turn, spotlights the role of “global ignition” in frontoparietal networks, and HOT—in all its variations (Richard et al. 2019)—posits that “higher-order” states in frontal areas play a key role in conscious processing.

These divergences between theories explain the large extent of the borderland and they also suggest big differences in how these theories operationalize consciousness. If the different theories would use the same methodology, it is very unlikely that they would produce such stable, but incompatible results. This methodological divergence was also tested by Yaron et al. (2022) who trained a classifier that could determine which theory was supported by a study based solely on methodological parameters (see also figures 4 and 5 in Yaron et al. 2022).

In summary, the operationalization problem has led to a wide range of candidate NCCs across the entire spatial and temporal domain. This presents a significant challenge for theory selection as it allows for a broad range of predicted NCCs to fall within the borderland. This lack of restrictions may explain why new theories continue to arise despite 30 years of research in the field.

Faced with this, the need to overcome disagreements is obvious. And there are basically two options that do not end in eliminativism about consciousness as a scientific phenomenon. The first option is to find ways to calibrate and validate a particular operationalization (Bayne and Shea 2020; Birch 2022). For example, by calibrating subjective reports which are assumed to be the gold standard to begin with (Spener 2015; Michel 2021), testing if markers and features of paradigm cases of consciousness do after all converge onto a single natural kind (Shea 2012; Bayne and Shea 2020), splitting consciousness up into different concepts (Block 2005; Dehaene et al. 2006, 2017; Pitts Michael et al. 2018), or pitching the explanatory power of theories against one another to justify a specific operationalization later on from a theory-heavy point of view (Lepauvre and Melloni 2021; Melloni et al. 2021; Del Pin et al. 2021).

The goal of this paper is not to evaluate these strategies, but to explore a second option, a backup plan of sorts, which may prove beneficial if these efforts turn out to be in vain. The idea is simple: even if scientists are unable to reach a consensus on how to precisely define or measure consciousness, it is still possible to agree on what consciousness is not. This backup plan enables us to continue the scientific investigation of consciousness, even when specific agreement is elusive. Such an approach serves as a bulwark against eliminativism and other forms of skepticism since it echoes methodologies already employed in other areas of scientific research, e.g. particle physics. The development and defense of the exclusionary approach will be the focus of the next part.

## From identification to exclusion

The logic of Baars’ contrastive analysis, aimed at defining the minimal conditions for the occurrence and non-occurrence of specific conscious experiences, remains a predominant force in NCC research. A significant drawback, however, emerges from this approach. The issue is not merely a demand for accuracy in measuring consciousness at near-threshold conditions, as all measurements inherently possess some level of inaccuracy. Rather, the crux of the problem resides in the absence of an agreed-upon operationalization to measure or detect consciousness under those conditions, which makes achieving a consistent level of accuracy across different contrastive studies elusive.

The lack of a shared operationalization forces researchers to embrace individualized definitions and methods, grounded in tentative assumptions. Such commitment yields divergence in interpretations and results, engendering a plethora of candidate NCCs with no discernible trends or consensus in the data, creating a considerable borderland of candidate NCCs. This fragmentation, notwithstanding efforts to refine and triangulate true NCCs, fosters skepticism and impedes advancement.

In the subsequent sections, I construct an argument anchored in a widely accepted notion that humans are good at comparing their vivid experiences under normal conditions. This foundation leads me to advocate for a novel approach in the field of NCC research that I term the “exclusionary” approach. Unlike the traditional strategy to identify NCCs by contrasting conscious and unconscious trials using near-threshold conditions, this approach leverages optimal conditions where report-based methods are deemed reliable even by skeptics. Here is how the methodology operates: it zeroes in on neural processes and events that systematically vary across two or more conditions, while concurrently using report-based methods to ensure that experiences remain stable across these conditions. By excluding these varying factors and their respective neural correlates from the pool of candidate NCCs, the exclusionary approach leverages report-based methods under normal conditions.

In the following sections, I will further elucidate the exclusionary approach and introduce three constraints that ensure the use of report-based methods in a credible and reliable manner, even for skeptics and despite differing views on how to operationalize consciousness.

### Why exclusion is better than identification of NCCs

Exclusion is widely accepted as a simpler process than identification, as it requires less information and fewer steps to reach a conclusion. For instance, excluding an individual based on their nationality or birth year is simpler than identifying them using specific features like fingerprints or previous visual recognition. By definition, exclusion involves the elimination of only one alternative, while identification inherently involves the elimination of all relevant alternatives. Exclusion is therefore less demanding than identification, but also a critical element of it. It is hard to see how identification can go without exclusion, stressing the complementary role of exclusion for identification. I argue that this reduction in demand means that the exclusionary approach can be used without operationalizing consciousness, making it a preferable strategy at the current stage of research.

The exclusionary approach contrasts somewhat with the oft-cited analogy between consciousness and temperature research (Irvine 2021; Michel 2021; Pauen and Haynes 2021). The prominence of this analogy follows directly from the goal of the standard approach. In order to contrast minimally different unconscious and conscious trials, we need a precise measuring instrument that can tell us exactly when conscious processing is instantiated and when it is not. This “consciousness meter” needs to be both sensitive and specific to consciousness. If we had one, we could easily correlate its precise readings with corresponding brain images and get a handle on the physical basis of consciousness (Chalmers 1998). The central question, however, is whether consciousness is sufficiently similar to phenomena like temperature for us to be able to operationalize it into a meter of consciousness.

In general, the process of operationalization involves transforming abstract concepts into tangible and quantifiable terms. In the case of temperature, this involves turning mean molecular movement or energy into a measurable value. The evolution of temperature measurement can be divided into four stages (Chang 2004; Irvine 2021). The first stage is the use of thermoscopes, which only indicate if something is hotter or colder than something else, but not the precise temperature. The second stage involves identifying fixed points that enable the comparison and calibration of different measuring instruments to these standard points. The third stage involves determining the relationship between the measurement scale of the instrument and the temperature between the fixed points. The final stage extends the measurement scale beyond the fixed points. This entire process is founded on two critical assumptions: realism and the principle of single value. Realism holds that temperature is a characteristic of the world and not a theoretical or experimental artifact. The principle of single value assumes that the property being measured has a unique value at any given time.

According to Irvine (2021), it is uncertain whether report-based methods can advance as a measure of consciousness beyond the first stage of operationalization. The reason is that there are no task-independent fixed points for introspective scales such as clarity or visibility. This means that the same stimulus can be perceived as clear or unclear depending on the task being performed, making it difficult to compare introspective reports across contexts. This variability may violate the principle of a single value. Without it, the validity and accuracy of a measure cannot be determined based on its repeatability. Irvine’s pessimistic conclusion is that a consensual operationalization of consciousness is unattainable (but see Michel 2021). I am going to take this conclusion for granted and consider how these difficulties can be bypassed.

Despite Irvine’s “dark pessimism” about consciousness as a scientific concept—culminating in eliminativism—she makes the following statement: “it seems reasonable to assume that introspectors are capable of thermoscope type measures, at least under normal conditions. That is, introspective agents are capable of telling, with a reasonable degree of validity and accuracy, when one experience is more clear than another, or when one stimulus is more visible than another” (Irvine 2021, 1329). This is the case even though we have no agreed-upon operationalization of consciousness. And even Schwitzgebel—a well-known skeptic—admits: “It is hard, seemingly, to go too badly wrong in introspecting really vivid, canonical pains and foveal colors” (Schwitzgebel 2011, 129). This kind of minimal optimism about reports is also in line with Bayne and Spener (2010), who agree that introspective judgments can be unreliable, especially when used outside their normal operating range. But they go on to argue that the skeptical threat of unreliable reports can be quarantined by focusing on introspective judgments that are “suitably scaffolded by perceptual judgments” (Bayne and Spener 2010, 20). It can be concluded that the use of “scaffolded” reports of “vivid” experiences under normal conditions as “consciousness scopes” leaves little room for skepticism.

The exclusionary approach builds upon this by imposing three constraints on the use of report-based methods. First, report-based methods should only be relied on as consciousness scopes rather than consciousness meters, i.e. to determine the presence or absence of a relative difference between experiences rather than an absolute value of consciousness. Second, report-based methods should only be used under normal conditions for vivid, canonical aspects of experience, rather than complex stimuli outside of attention or near-threshold conditions. Third, report-based methods should be scaffolded by perceptual judgments, allowing for some degree of objective assessment. The aspects of consciousness that are not scaffolded by a perceptual judgment, such as cognitive phenomenology, dreams, and emotions, are not (at this stage) considered.

The constraints outlined earlier present a hurdle to the standard approach, which seeks to determine the precise onset of conscious processing in absolute terms. Therefore, we must be able to either measure consciousness, which implies quantification on an agreed-upon scale, or detect it, which involves determining its presence or absence. Much like a thermostat that records a specific temperature or a metal detector that detects the presence or absence of metal, but for consciousness. This requires the aforementioned tool, metaphorically referred to as “consciousness meter.” However, reliable measures or detection rules often falter when inverted. For example, the absence of a report does not clearly equate to the absence of consciousness, and performance above chance does not consistently confirm the presence of conscious processing. To explore the nuances of the constraints and their friction with the standard approach, let us examine each of them in more detail:

- Consciousness scope: The standard approach strives to determine the minimal conditions that mark the presence and absence of consciousness. A consciousness scope, as discussed here, is ill-equipped for this task, as it only offers relative assessments of conscious experiences, not absolute measurements. Consequently, it falls short in isolating and identifying these minimal conditions as it cannot pinpoint trials that differ only in terms of consciousness.
- Vividness: This constraint promotes the use of vividly experienced phenomena for report-based methods. However, the standard approach, to isolate consciousness as a variable, demands precision under less vivid, near-threshold conditions. This involves complex or subtle stimuli that do not induce the unequivocal, vivid experiences that are reported with high confidence. Moreover, the notion of a “vivid unconscious experience” seems almost paradoxical, as the term “vivid” is typically associated with consciously present experiences. However, there could be other ways to leverage clear instances of unconscious processing.
- Scaffolding: This constraint requires that report-based methods be underpinned by perceptual judgments. On the surface, this requirement seems in line with the standard approach, yet the complexity lies in its practical implementation. While the standard approach does not inherently demand perceptual scaffolding, it can certainly accommodate it.

To reconcile these constraints with the study of consciousness, a shift from identification to exclusion is necessary. Candidate NCCs can still be excluded through contrastive analysis as demonstrated by distilling paradigms. By using report-based methods only to testify that the relevant aspects of experience remain consistent across conditions while manipulating another variable (reports, expectations, attention, and stimulation), the impact of that manipulated variable on brain activity can be measured. Since the observed differences in neural activity are not the result of changes in experience, they can be excluded as candidate NCCs. This method is feasible and produces less controversial results compared to attempts to identify or distill true NCCs. The recognition that report-based methods can serve as reliable consciousness scopes, even among skeptics like Irvine and Schwitzgebel, lends credibility to the belief that the exclusionary approach can establish a widely agreed-upon foundation.

Finally, it is worth mentioning that exclusionary approaches can be found in other scientific disciplines. While many fields, such as the study of light spectrography to identify the elemental compounds of a gaseous planet, focus on identification, others, in particular particle physics, use exclusion to make progress. Instead of directly identifying a fundamental entity, scientists work toward it by eliminating non-fundamental entities by breaking them down into subcomponents. This is how we have arrived at the current understanding that quarks are fundamental particles, at least for now. “There is speculation that if you could magnify an electron or a quark another billion billion times, you would discover the underlying Morse code to be like strings” (Close 2004, 5). This illustrates that it is not always necessary to have a concrete operationalization of a theoretical concept in order to study it in science. The exclusionary approach is widely considered to be a mature and valid scientific approach, at least in physics. To summarize, perhaps consciousness science is more like particle physics and less like the invention of temperature.

In the following sections, I demonstrate how each of the constraints presented makes distilling paradigms resistant to the most frequently raised criticisms. I use no-report paradigms as a proof of concept for the exclusionary paradigms (distilling under constraints) to establish consciousness research as a proper scientific project even if the operationalization problem remains unresolved.

### The consciousness scope constraint

The preliminary no-report study by Frässle et al. (2014) explored the neural mechanisms behind initiating perceptual alternations during binocular rivalry using fMRI. In the study, one eye was presented with a grating moving to the left and the other with one moving to the right. To objectively measure when perception alternates, the researchers used a reflexive eye movement known as optokinetic nystagmus (OKN), which has been shown to be linked to the motion of the currently perceived image. This was confirmed by the fact that the direction of the OKN reliably predicts the reported direction. This enabled the creation of two conditions: an active one, in which switches were mapped using active reports, and a passive one, in which the OKN was used.

The study first isolated rivalry-related activity by contrasting rivalry with replay conditions and then contrasted rivalry-related activities in active and passive trials. The fMRI scans revealed substantial differences between the active and passive conditions, with a near-total reduction in rivalry-related frontal activity during passive viewing. The authors concluded that this difference in report-related frontal activity is linked to active reporting or introspection, rather than being the driving force behind perceptual alternations in binocular rivalry. However, interpreting this differential rivalry-related activity in the context of consciousness proves challenging.

There are two prominent conclusions one might draw from this study. The first is implied in the paper’s title, “Frontal Activity Relates to Introspection and Action But Not to Perception.” This title suggests that there are no NCCs of conscious perception in frontal areas but only report-related activity. This interpretation of the data, however, is highly problematic (see Michel and Morales 2020; Michel 2022b).

Note that, in the active condition, the differential activity depicts the relative increase in rivalry compared to replay conditions, even though participants report stimuli in both situations. This suggests that the differential activity in the active “report” conditions is not actually attributable to reporting, as any activity directly associated with it is effectively canceled out by contrasting reported rivalry with reported replay. Passingham (2021) proposes an alternative interpretation: the heightened report-related activation seen in active as opposed to passive conditions could stem from the increased challenge participants face when deciding when to report during rivalry, compared to replay conditions. Furthermore, the merely subtle differences in conscious perception between rivalry and replay conditions suggest that parts of the proper NCC are canceled out as well. In fact, it is far from clear that we can associate rivalry-related activity with NCCs at all. The first conclusion that frontal activity is merely reflective of the reporting process itself, but not of consciousness, is an oversimplification. The conclusion that there are no NCCs in frontal areas is quite contentious.

In line with the exclusionary approach, the more lenient but credible conclusion is to exclude the specific differential activity between active and passive conditions as a candidate NCC, considering that experience remains stable across both conditions. Since the differential activity is better explained by the variant factor—active vs. passive—than by constant consciousness, the dissociation is evident. However, this does not rule out the entire neural population in the frontoparietal areas from playing a role in consciousness. While the differential activity can be dissociated from consciousness, other neural events in the same population might still be part of the proper NCC. Hence, the exclusionary approach must be careful to focus on specific ERPs rather than dismissing entire regions, recognizing that only the differential activity in exclusionary paradigms can be explained away by the variant factor rather than an entire region. Our primary interest lies in this second conclusion: there exists a significant differential activity pattern in the frontoparietal areas that can be excluded as an NCC because it is associated with a variant factor in the face of relatively stable experiences across conditions.

This second conclusion has been supported by numerous more recent studies (e.g. Sergent et al. 2021; Elaheh et al. 2022; Vishal et al. 2022). The locus of the difference between active and passive conditions was specified on an ERP known as P3b, which is characterized by a positive spike in activity over frontoparietal areas ∼300 ms after stimulus onset. The absence of P3b in passive no-report conditions has been consistently demonstrated across various paradigms and modalities, calling into question long-held theories that posit P3b over the frontoparietal network as a primary candidate NCC (Dehaene and Changeux 2011). Its exclusion thus would make a significant contribution to the field. However, some still claim that there is no consensus yet on whether P3b is a confound or a proper NCC (Mashour George et al. 2020). In the following, three major critiques against the conclusiveness of these no-report results are presented and neutralized using the three constraints.

The first criticism of no-report paradigms challenges the claim of distilling the “true” NCCs as stated by Naotsugu et al. (2015). Overgaard and Fazekas (2016) argue that no-report paradigms are just as susceptible to confounding factors as report paradigms and therefore still lead to an overinclusion of confounding processes into the measured NCCs. They point out that switches in binocular rivalry include many stages of processing, including pre- and post-conscious ones. For instance, the OKN likely tracks pre-conscious events such as retinal image stabilization or norepinephrine release. Furthermore, no-report paradigms are not immune to confounds related to post-conscious events, such as either introspecting on or attending to the alternating contents of experience. Thus, it is important to acknowledge the limitations of no-report paradigms when interpreting results as a distillation of true NCCs.

So how does the goal of identifying the true NCCs align with the consciousness scope constraint? Consciousness scopes can only provide a subjective indication of the relative difference between experiences, rather than a precise, objective measurement of the level of consciousness. This means that consciousness scopes can be used to determine if an experience remains stable across trials or changes, but cannot be used to identify a clear transition point between unconscious and conscious processing. Given these limitations, it is important to reconsider the use of no-report or similar paradigms as a means to isolate the true NCCs, as this objective cannot be achieved without relying on report-based or other methods such as consciousness meters. Which is equal to solving the operationalization problem. Through this lens, studies that dismiss the P3b as a candidate NCC (Frässle et al. 2014; Pitts Michael et al. 2014; Sergent et al. 2021; Elaheh et al. 2022; Vishal et al. 2022) are interpreted not as endeavors to distill “true” NCCs, but primarily as paradigms aiming to exclude confounding factors.

The no-report paradigm used by Frässle and colleagues now contrasts two sets of rivalry-related activity. One set uses active reports to mark switches, while the other uses OKN. By doing so, the authors compare rivalry-related activity with and without report-related activity. Note again that report-related activity is not strictly related to reporting, as this activity has already been canceled out using replay conditions as a contrast to rivalry. Now, making the claim that the report-related activity is not an NCC is distinct from asserting that it is associated with reports. Moreover, it is quite different from suggesting that the remaining rivalry-related activity in passive no-report conditions distills the true NCC. This latter point in particular requires every perceptual switch to accurately delineate the minimal conditions differentiating conscious from unconscious processing. This assumption disregards the intricacies involved in rivalry switches. Without a perfect consciousness meter, true distillation cannot be achieved in a single step. Rather, a systematic and stepwise approach must be taken to eliminate confounds one by one to account for the issue of operationalization.

In summary, the consciousness scope constraint is a useful counter to overinclusion critiques as it emphasizes the exclusion of candidate NCCs rather than the identification or distillation of true NCCs. This is incremental in shaping our interpretations of studies in consciousness research toward emphasizing exclusion while remaining vigilant toward any oversimplified identification of true NCCs. It stresses an exclusionary lens, so to speak. Moreover, it is crucial to remember that many investigations spurred by the confounding factors problem do not aim at pinpointing the “true” NCCs. Instead, they seek to expose clear instances of confounding factors, thereby aligning with consciousness scope constraint. Notably, these include studies that dismiss the P3b ERP as a legitimate NCC candidate (Frässle et al. 2014; Pitts Michael et al. 2014; Sergent et al. 2021; Elaheh et al. 2022; Vishal et al. 2022). Hence, the exclusionary approach not only serves to combat overinclusion but is also in line with an effective framework for disentangling confounding variables in the quest to narrow down the borderland of the neural basis of consciousness.

### The vividness constraint

“What’s wrong with the no-report paradigm?” is the question posed by Block (2019) and it refers to the same criticism discussed in the previous section. This section will not revisit the criticism, but examine Block’s proposed solution. The main concern raised by Block is the “bored monkey” problem. It occurs when participants are exposed to stimuli for an extended period without a task to perform, as is often the case in no-report conditions [some recent studies add low-intensity tasks to no-report paradigms because of that (e.g. Cohen et al. 2020)]. In such a scenario, participants may engage in cognitive processes that align with one of the conflicting images. Block is not referring to mind-wandering or daydreaming, but to systematic cognitive processes that follow one of the conflicting images. For instance, some cognitive processes might be more reliably triggered by a movement to the left than by a movement to the right. Block argues that these systematic post-perceptual cognitive processes prevent no-report paradigms from uncovering the true NCCs and may explain why some studies (e.g. Lumer and Rees 1999) still observe differential activity in frontal areas in the absence of reports.

Block’s solution to the bored monkey problem is to replace no-report paradigms with “no-cognition” paradigms, a method he says has already been successfully implemented in a study by Brascamp et al. (2015). Their goal was to design binocular rivalry switches that were “inconspicuous,” making perceptual alternations during rivalry unnoticeable to subjects. By presenting each eye with different patterns of moving dots changing direction every 300 ms, an impression of rapid and irregular shifts of global motion was created. When the dots were colored differently, subjects easily detected rivalry switches. However, when the dots were the same color in both eyes, detection rates dropped to near chance level, with changes in experience due to rivalry switches indistinguishable from the objective jitter occurring every 300 ms. Frontal activity related to these unnoticeable switches was deemed “negligible.”

Block takes this as evidence of binocular rivalry where experience alternates without the subject noticing, and negligible involvement of frontal areas leads him to conclude that these areas play no role in experiencing but only noticing changes in experience. If Block’s interpretation holds, it implies exclusion of frontal candidate NCCs, since experience can alter independent of frontal activity. As cognitive access is often linked to these areas, this could mean consciousness is a non-cognitive phenomenon, underlining the significance of this interpretation.

Two alternative interpretations challenge Block’s claim that frontal areas do not play a role in conscious perception, especially considering the complexity of the moving dot stimuli used in the study. It is important to note that the contrast image showing negligible activity results from subtracting intervals with changes due to a shift in dominance from intervals in-between where the dots physically changed direction. Determining what truly differed between the two intervals in terms of perceiving and noticing is difficult, and this uncertainty motivates the need for a vividness constraint or a focus on clearer stimuli.

First, rivalry switches in same-color conditions may have been noticed, but in the same way, objective jitters were noticed. This underscores that the study’s design might not fully eliminate post-perceptual cognition in the same-color conditions, but only the post-perceptual cognition that differs between objective and rivalry-induced changes, as pointed out by Phillips and Morales (2020). In this scenario, noticing would accompany all changes in experience—both rivalry and jitters—but subjects are unable to discern they have different causes.

Second, another rather speculative interpretation could posit that changes are not noticed because they are not experienced, suggesting genuinely invisible rivalry switches. This interpretation hinges on the question of whether global motion is something we truly perceive or an illusory aspect, potentially embedded into our experience by statistical decision-making processes. The far-from-vivid stimulus adds to the challenge of discerning what is actually experienced and what is illusory.

Together, both interpretations cast doubt on the conclusion that noticing and experiencing are dissociated. Thus, the absence of differential activity does not necessarily indicate a lack of involvement of frontal areas in changes of experience. Either it plays the same role in both objective jitters and rivalry changes, or the lack is unproblematic, as experience remains relatively stable, with only the cognitive illusion of global motion direction altering.

In conclusion, Brascamp and colleagues may indeed have confirmed that frontal areas play no causal role in initiating rivalry switches, but they have not dissociated frontal activity from changes in experience. Block’s interpretation seems to beg the question against advocates of the illusory richness of experience, such as Kouider et al. (2010), if applied to global motion, or against those wary of conflating the processes that cause rivalry switches with the property of being an NCC of the resulting changes in experience (Phillips and Morales 2020). Specifically in line with the latter, a study by Zou et al. (2016) implies that rivalry can occur even for entirely invisible stimuli, suggesting that rivalry mechanisms are indeed pre-conscious processes. If so, the rivalry-related activity in the same-color condition is itself not part of the NCC of the resulting changes. Along with this, it is essential to keep in mind that the jitters in the random dot stimulus were strategically designed to mimic and mask the changes in experience during rivalry switches.

In summary, although there are valid concerns about interpreting the content of experience in no-cognition paradigms, the vividness constraint alleviates these concerns by emphasizing that we use the same stimuli across conditions that elicit vivid and canonical experiences. This enhances the reliability of respective subjective reports, testifying that experiences remain relatively stable across different conditions, such as report vs. no-report. It also sidesteps debates over potentially misleading or illusory aspects of the experience, including its richness. It is worth reiterating that many studies identifying P3b as a confound adhere to this constraint, utilizing the same simple stimuli—such as constantly moving gratings, single vowels, or common objects—in both conditions (Frässle et al. 2014; Pitts Michael et al. 2014; Sergent et al. 2021; Elaheh et al. 2022).

### The scaffolding constraint

The final criticism of no-report paradigms is a less commonly raised issue in the literature, but a straightforward one. It suggests that the contrast between report and no-report conditions may actually be, at least in part, an NCC and is therefore “underinclusive” (Duman et al. 2022). Duman and colleagues argue that the contrast between these conditions may exclude the neural correlates of conscious disengagement, which is described as a “qualitative change in consciousness, in which it turns inward and engages in daydreaming and mind-wandering” (Duman et al. 2022). This is a problem because the contrast between no-report and report conditions could itself be a candidate NCC. To use this contrast to exclude the underlying activity as a candidate NCC would thus be incorrect. This issue can easily be generalized to task-dependent cognitive and epistemic aspects of experiences. For example, confidence, agency, or confusion are all feelings that are dependent on the task being performed and therefore may be excluded as candidate NCCs if the contrast between report and no-report conditions is used.

A way to avoid the issue can be found by examining the difference between two types of reports as introduced in Bayne and Spener (2010): scaffolded and freestanding ones. They put it this way: “A scaffolded judgement is an introspective judgement about a conscious state P the content of which matches closely the content of a different judgement the subject would be disposed to make when endorsing the content of P” (Bayne and Spener 2010). In contrast, freestanding judgments are those that are not scaffolded. Many of the introspective judgments that we naturally regard as trustworthy are strongly scaffolded, usually by perceptual judgments with the same content. For example, if you introspectively judge that it looks to you as if there is coffee in front of you, and you make a congruent perceptual judgment that there is coffee, the former is scaffolded by the latter. The content similarity also allows reliability to leak from the perceptual to the introspective domain allowing for an objective assessment, i.e. you gain further confidence in your introspective judgments if there is indeed coffee. On the other hand, introspective judgments made in the context of cognitive phenomenology are much riskier because they are freestanding or weakly scaffolded and cannot be checked against any first-order perceptual judgments.

This provides another way to approach the problem. The limitations of no-report and distilling paradigms in capturing neural correlates of some cognitive and epistemic aspects of experience should not be discouraging. These aspects of experience cannot be reliably tested against perceptual judgments and may require a different approach. However, focusing on the aspects of experience with content similar to objectively accessible perceptual judgments is crucial for assessing introspective accuracy. As Bayne and Spener (2010) argue, introspective judgments are more likely to be correct if they align with veridical perceptual judgments. Therefore, studying perceptual or sensory aspects of experience under conditions where reports and perceptual judgments align can increase the likelihood of accurate introspective judgments. This motivates to restrict research on perceptual or sensory aspects of experience only for the moment.

This also highlights the importance of being transparent about the specific aspects or contents of experience being tested when using no-report paradigms to exclude candidate NCCs. Differentiation between NCCs based on content is a widely accepted practice in the field of consciousness research (Koch et al. 2016; Boly et al. 2017) and has proved to be important in relation to estimate the extent of unconscious perception (Kahneman 1968; Michel 2022a).

In summary, the exclusionary approach recognizes that no-report and other distilling paradigms may be underinclusive, but this limitation is countered by the focus on those aspects of experience that are scaffolded by perception. This allows for a more reliable assessment of the aspects of experience under investigation and highlights the importance of content specificity. Again, many studies pointing out that P3b is a confound adhere to this constraint because they concentrate on perceptual aspects of experience (Frässle et al. 2014; Sergent et al. 2021; Elaheh et al. 2022; Vishal et al. 2022).

## Conclusion

The exclusionary approach tackles skepticism in the study of consciousness by shifting NCC research from identification to exclusion. This shift stresses distilling paradigms to adhere to three constraints: the consciousness scope constraint, the vividness constraint, and the scaffolding constraint. By following these constraints, researchers can create exclusionary paradigms that bypass the need to agree on an operationalization of consciousness and mitigate disputes about the richness of experience and its rather elusive cognitive or epistemic aspects. This approach only uses report-based methods as consciousness scopes to assess the relative stability of the vivid perceptual aspects of experiences, thereby making it difficult to deny their reliability even for dark pessimists and skeptics. The excluded candidate NCCs have been dismissed on a consensual basis and establish a minimal common ground of hard criteria for theories of consciousness. Theories that predict already excluded candidate NCCs need to be adapted or rejected.

Influential studies, including those by Frässle et al. (2014), Sergent et al. (2021), Elaheh et al. (2022), and Vishal et al. (2022), have shown adherence to the three constraints when interpreted within the boundaries of the exclusionary approach. This leads to the targeted exclusion of a specific differential neural activity pattern in the frontoparietal areas (P3b) as a candidate NCC of perceptual experience. Theories of consciousness that predict P3b to be an NCC, such as many versions of the GWT (e.g. Dehaene and Changeux 2011), need to be adapted or rejected in light of this evidence. Making progress in this regard also complements our initial efforts to identify the proper NCCs and might result in fewer candidate operationalizations as we successively narrow down the borderland of candidate NCCs.

It is important to note that it could be that the neural population in frontoparietal areas driving the P3b also realizes another ERP in the 100 or 200 ms range that is a proper NCC. The very neurons that realize this NCC might be modulated in report conditions due to interactions with read-out systems, resulting in the observable P3b pattern that can be dissociated from consciousness. But the fact that it is an NCC population being modulated is compatible with the exclusion of the respective differential activity. Such modulation might be essential for the experience to be reported, but considering a stable experience across trials, it is not part of the minimally sufficient set of neural events required for instantiating the experience. This highlights that excluding an entire neural population from playing a role in consciousness is much more challenging. The exclusionary approach should thus focus on specific ERPs, rather than entire regions.

There is also a natural flipside to the paradigms we have discussed. Conditions where participants have no conscious awareness of stimuli, even under the most conservative measures, might provide another window to exclude certain processes as candidate NCCs. There are two specific logics that can be employed within this flipside.

The first logic is straightforward. If neural activity is associated with a completely unconscious stimulus, it should be excluded as a candidate NCC. But caution is warranted, as this might inadvertently exclude genuine parts of the proper NCCs that only require an additional factor to become conscious.

The second logic explores maintaining a stable experience while altering preceding processes, like physical stimulation. For example, comparing the perception of a green disk with an identical one created from flickering yellow and red disks (Zou et al. 2016) falls under this logic. However, this approach has its own intricacies; changing the processes preceding consciousness might impact the proper NCCs, as shown by Melloni et al. (2011), who found that expectations could shift the timing of candidate NCCs from the 300 ms range to the 200 ms range.

Therefore, while no-report studies and other exclusionary paradigms offer exciting prospects for exploring and ruling out candidate NCCs, vigilance must be exercised from case to case, and more constraints may be needed.
